# Development and evaluation of ^89^Zr-trastuzumab for clinical applications

**DOI:** 10.22038/AOJNMB.2022.68093.1471

**Published:** 2023

**Authors:** Fatemeh Mohammadpour-Ghazi, Hassan Yousefnia, GhasemAli Divband, Samaneh Zolghadri, Behrouz Alirezapour, Fatemeh Shakeri

**Affiliations:** 1 Radiation Application Research School, Nuclear Science and Technology Research Institute, Tehran, Iran; 2 Khatam PET/CT Center, Khatam-Al-Anbia Hospital, Tehran, Iran; 3 Department of Clinical Biochemistry, Faculty of Medical Sciences, Zanjan University Medical Sciences, Zanjan, Iran

**Keywords:** ^89^Zr, Trastuzumab, HER2 positive tumors, Breast cancer

## Abstract

**Objective(s):**

Due to the suitable physical characteristics of ^89^Zr as a PET radionuclide and affinity of Trastuzumab monoclonal antibody against HER2, [^89^Zr]Zr-Trastuzumab was prepared and went through preclinical evaluations for ultimate human applications.

**Methods:**

^89^Zr was produced by using ^89^Y(p,n)^89^Zr reaction at a 30 MeV cyclotron (radionuclide purity>99.9%, specific activity of 17 GBq/µg). p-SCN-Bn-Deferoxamine (DFO); was conjugated to trastuzumab, followed by labeling with ^89^Zr in oxalate form at optimized condition. Cell binding, internalization and, radioimmuno-activity assays were studied using HER2+ BT474 and HER2- CHO cell lines. Finally, the biodistribution of the radioimmunoconjugate was assessed in normal and HER2+ BT474 tumor-bearing mice using tissue counting and imaging at different intervals after injection. Also, a woman with HER2-positive metastatic breast cancer under treatment with Herceptin underwent both [^89^Zr]Zr-Trastuzumab and, [^18^F]FDG PET/CTs.

**Results:**

^89^Zr was produced with high radionuclidic and radiochemical purities (>99%) and [^89^Zr]Zr-DFO-Trastuzumab was prepared with radiochemical purity of >98% and specific activity of 9.85 GBq/µmol. The radioimmunoconjugate was stable both in PBS buffer and in human serum for at least 48 h. The radioimmunoactivity assay demonstrated about 70% of [^89^Zr]Zr-DFO-Trastuzumab is bound to the BT474 cells at the number of 250×10^6 ^cells. Cell binding studies showed that about 28% of radioimmunoconjugate is attached to BT474 cells after 90 min. Internalization studies showed that 50% of [^89^Zr]Zr-Trastuzumab is internalized to BT474 cells only in 6 h. The biodistribution study of the labeled compound in normal mice demonstrated the same pattern of the monoclonal antibodies which is entirely different from the biodistribution of free ^89^Zr. Biodistribution and imaging studies in tumor-bearing mice showed the significant uptake values of [^89^Zr]Zr-Trastuzumab in tumor sites. [^89^Zr]Zr-Trastuzumab PET/CT revealed metastatic lesions documented previously with [^18^F]FDG PET/CT scan in a woman with breast cancer who was under treatment with Herceptin. Although the [^18^F]FDG PET/CT scan had better quality images, the valuable and unique advantage of [^89^Zr]Zr-Trastuzumab PET/CT is delineating HER2+ metastasis, which is essential in diagnosis and HER2-based treatments.

**Conclusion:**

The prepared [^89^Zr]Zr-Trastuzumab has a high potential radio-pharmaceutical for immune-PET imaging of the patients with HER2+ tumors.

## Introduction

 The amplification or overexpression of human epidermal growth factor 2 (HER2) in 15-30% of breast and 10-30% of gastric/gastroesophageal cancers as well as the other types of cancers, including ovarian, endometrium, bladder, lung, colon, prostate and head and neck, made it an attractive biomarker for diagnostic and therapeutic purposes (1, 2). Trastuzumab as a monoclonal antibody tyrosine kinase inhibitor targeting HER2 has been indicated good efficacy as a single agent or in combination with chemotherapy in patients with HER2-positive metastatic breast cancer(3).

 While nuclear medicine indicated substantial function in breast cancer imaging, numerous radiolabeled compounds have been introduced in this scope (4, 5, 6 ) such as 16α-[^18^F]-fluoro-17β-estradiol (FES) and the radiolabeled monoclonal antibodies (MoAb) such as trastuzumab as attractive radiopharmaceuticals (4). Whereas radiolabeling of trastuzumab with different radionuclides has been reported (7, 8, 9), ^111^In-labeled trastuzumab indicated accurate detection of HER-2-positive tumors in patients with breast cancer (7). 

 In recent years, special attention has paid to the production of antibody-based ^89^Zr radiopharmaceuticals thanks to its favorable decay characteristics (T1/2=78.41 h, EC=76.6%, β+=22.3%, Emax(β+) =897 keV, Eave(β+) =397 keV, Rave(β+) =1.18 mm, Eγ =908.9keV, Iγ =100%) as well as the compatibility of its physical half-life with the biological half-life of antibodies (10, 11 ). Until now, different clinical trials for PET imaging based on ^89^Zr radiopharmaceuticals have been reported utilizing trastuzumab, bevacizumab, cetuximab, rituximab, NMOTO53OA, ibritumomab-tiuxetan, cmAb U36 and, Hu-J591 MoAb (12). 

 The preliminary studies on [^89^Zr]Zr-Trastuzumab in nude mice bearing HER2-positive tumors showed high tumor accumulation and a similar biodistribution pattern with ^111^In-trastuzumab (13). The first clinical trial of [^89^Zr]Zr-Trastuzumab (2010) in 14 patients with metastatic breast cancer indicated its potency for better diagnosis of HER2-positive breast cancer (14). The results also showed the ability of [^89^Zr]Zr-Trastuzumab immune-PET imaging to detect metastatic liver, lung, bone, and even brain lesions in patients with HER2-positive breast cancer. The recent study on the absorbed dose estimation of [^89^Zr]Zr-Trastuzumab represented its safety, while the liver is recognized as the dose-limiting organ(15). 

 Despite the promising results of this radiolabeled compound in diagnosing HER2-positive tumors, more studies are still needed, especially for entirely in-house productions. This study was aimed to explain the preparation and quality control studies for an in-house produced ^89^Zr-DFO-Trastuzumab. In contrast, the in-vitro cell studies on BT474 cells as well as the biodistribution in tumor-bearing mice, were also evaluated. 

## Methods

 ZR resin, trastuzumab and p-SCN-Bn-Deferoxamine (DFO) were provided from TrisKem International Co. (France), Ariogen Pharmed Co. (Iran), and Macrocyclics Inc. (USA), respectively. Yttrium oxide powder (with chemical purity of 99.9%) and all other chemical reagents were purchased by Sigma Aldrich Co. 30 MeV cyclotron (Cyclone-30, IBA) was utilized to prepare ^89^Zr from ^89^Y(p,n)^89^Zr reaction. Radiochemical purity and radio-nuclidic purity were investigated using bio scan AR-2000 radio TLC scanner instrument (Bio scan, Paris, France) and, a p-type coaxial high-purity germanium (HPGe) detector (model: EGPC 80-200R), respectively. Calculations were performed based on the 908.9 keV peak of ^89^Zr. HER2+ BT474 cell lines were supplied from Pasteur Institute, Iran. Nude mice were prepared from Royan Institute, Iran. The animal experiments were performed according to the NIH animal use and care guidelines. Images were acquired using a dual-head gamma camera system (model: DST-XL made by SMV company). The biodistribution data were compared using Student’s T-test while the statistical significance was defined as P<0.05. The human study was conducted with the ethical approval code of IR. BPUMS.REC. 1401.025. 

***Production and Quality Control of*** ^89^ ***Zr***

^89^Zr was produced by the bombardment of ^89^Y_2_O_3_ pellet target with 15 MeV proton energy for 5 h while the current was 25 µA. The target was dissolved in 6 M HCl solution. ZR resin (TrisKem) was utilized to separate the ^89^Zr from the natY target and the other possible impurities. About 200 mg of ZR resin was packed as a separation column for 0.33g of ^89^Y oxide pellet. 6M HCl was applied for conditioning the column. After washing four times with 2.5 mL HCl solution and four times with 2.5 mL water, ^89^Zr was obtained in 1.5 mL of 1.0 M oxalic acid solution. The radiochemical purity of the product was determined by the RTLC method using Whatman paper chromatography as the stationary phase and 20 mM citric acid as the mobile phase. The radionuclidic purity was investigated by an HPGe detector considering the gamma peak energy of 908.9 keV. Chemical purity was assessed by ICP-Mass.

***Conjugation of DFO with Trastuzumab***

 The conjugation procedure was carried out according to the previously reported study with some modifications (16). Briefly, DFO was dissolved in DMSO, and trastuzumab (5 mg/mL) was conjugated to a molar excess of DFO at 37°C for 30 to 90 min. The reaction pH was adjusted to 9.0 with sodium carbonate 0.1 M. Two separate experiments were designed to optimize the chelator: mAb (3:1 and 10:1) ratios. To investigate the optimal time for conjugation, different intervals were considered. On the other hand, there was an additional optimization on the conjugation time. The prepared conjugate was purified using PD-10 column prewashed with 5 mg/mL gentisic acid in 0.25 M sodium acetate. After transmission of the conjugation mixture, the column was washed twice with 1.5 mL of 5 mg/mL gentisic acid in 0.25 M sodium acetate and the final solution was collected as the purified antibody for radiolabeling purposes. 

***Labeling of DFO-Trastuzumab with*** ^89^ ***Zr***

 Although the previous study was considered an appropriate method for the labeling step (16), numerous experiments were performed considering the different reaction parameters (^89^Zr: mAb ratios, pH, and time) to optimize the radiolabeling conditions. Briefly, 200 µL of 1 M oxalic acid and 100 µL of 2 M Na_2_CO_3_ were added to a vial containing 37 MBq [^89^Zr]Zr-oxalic acid. While, shaking the reaction vial, different amounts of conjugated trastuzumab (0.5-2 mg) and HEPES buffer was poured. The effect of temperature (20°C to 35°C), time, and pH in radiolabeling efficiency were considered. Eventually, PD-10 column was utilized to access to the purified radiolabeled compound. The radiochemical purity was investigated by the RTLC method using Whatman paper as the stationary phase, and 20 mM citric acid (pH=4.5) as the mobile phase.

***Stability studies of [*** ^89^ ***Zr]-Trastuzumab***

150 µL of the radiolabeled compound was added to the 500 µL of the PBS buffer and freshly prepared human serum and the temperature was set to 4°C and 37°C, respectively. The radiochemical purity was checked out at several intervals by the deployment of the ITLC method (2, 4, 24, and 48 h after preparation). 

***Cell cultures***

 The HER2+ BT474 and CHO cell lines were commercially provided and grown in a monolayer 75 cm^2^ tissue culture flask in RPMI 1640 medium supplemented with 10% heat-inactivated fetal bovine serum, 100 μg/mL streptomycin and 100 IU/ml penicillin.

***Immunoreactivity of the radioimmunoconjugate towards HER2+ BT474 and CHO cell lines***

 The immunoreactivity of [^89^Zr]Zr-Trastuzumab toward its relevant antigen (HER2) was assessed according to the LINDMO method (17). 

 Briefly, HER2+ BT474 and CHO cell lines were seeded in a 75 cm2 tissue culture flask and were kept at 37°C overnight. A serial dilution of BT474 and CHO cells, starting at 5×10^6^ cells/mL, including 5×10^6^, 2.5×10^6^, 1.25×10^6^, 0.6×10^6^, 0.3×10^6^ cells/mL was obtained and was mixed with 30,000 cpm of the ^89^Zr-DFO-Trastuzumab. To saturate the binding sites, the unlabeled trastuzumab (50 ng) was added to microcentrifuge tubes. ^89^Zr-DFO-Trastuzumab (50 ng/mL) was added to the tubes. The tubes were incubated overnight at 4°C while gently shaking. Following to this step, by the help of a γ-counter the radioactivity of each tube was measured. Afterwards the tubes were centrifuged in 3000×g, the supernatant was disposed and respective radioactivities of the cell debris were measured by means of γ-counter. The immunoreactivity was ascertained by making use of a Lineweaver-Burk plot and Lindmo method was applied to analyze the data.

***Binding studies***

 As a cell uptake experiment, the binding assay was performed in HER2+ BT474, and HER2- CHO cells. For this purpose, 0.250×10^6^ cells were seeded in 24-well plates and incubated overnight at 37°C. The new media (250 μL), including 37 KBq of [^89^Zr]Zr-Trastuzumab (50 ng/mL), was added to the well after removing the media, and the plates were incubated at 4°C for 1.5 h with mild shaking. The cells were trypsinized and transferred to tubes. 

 Radioactivity associated with cells was counted in a gamma counter. The percentage of bound radioactivity was calculated as the ratio of intent to the total radioactivity added per well multiplied by 100. This procedure was repeated three days after the first binding assay. 

***Internalization***

 Internalization assay was carried out according to Marquez et al. report with some modifications (18). Briefly, 1×10^6^ cells were seeded in 12-well plates and incubated overnight at 37°C. The new media (250 μL) including [^89^Zr]Zr-Trastuzumab (50 ng/mL) was added to the well after removing the media, and plates were incubated at 37°C for 1, 3, 6, 24, 28 and 48 h. As a control, the reactions were 

repeated for CHO cells. The cells were washed three times, and trypsinized to dissociate from the well and transferred to the microcentrifuge tubes and sedimented at 6000g. After supernatant collection, 100 μL of 0.1 M sodium citrate (pH=2) was added to the cells and incubated for 5 min. The cells were sedimented at 6000g, and counted. The amount of ^89^Zr-mAb bound on the cell surface and internalized was calculated.

***Tumor implantation***

 Nude mice (4 to 8 weeks old, 20-25 g weight) were provided from Royan Institute, Iran. About three million HER2+ BT474 cells were counted and injected subcutaneously into the nude mice. The biodistribution and imaging studies were carried out after the tumor had grown up to 7‑8 mm^3^.

***Biodistribution studies of [*** ^89^ ***Zr]Zr-Trastuzumab in normal and tumor-bearing mice***

 1.85MBq of ^89^Zr-DFO-Trastuzumab was injected into the tumor-bearing nude mice. The mice were sacrificed at the specified time after injection while the activity of each tissue was determined using an HPGe detector according to Equation 1 (IAEA, 2004):

A=Nϵ γ ts m k1 k2k3 k4 k5 (1) 

 Where ε is the efficiency at photopeak energy, γ is the emission probability of the gamma line corresponding as the peak energy, is the life time of the sample spectrum collection in seconds, m is the mass (kg) of the measured sample, $k_{1}$, $k_{2}$, $k_{3}$, $k_{4}$ and $k_{5}$ are the correction factors for the nuclide decay from the time the sample is collected to start the measurement, the nuclide decay during counting period, self-attenuation in the measured sample, pulses loss due to random summing and the coincidence, respectively. N is the corrected net peak area of the corresponding photopeak given as:

N=Nsts tb Nb (2)

 Where$N_{s}$ is the net peak area in the sample spectrum, $N_{b}$ is the corresponding net peak area in the background spectrum, and$t_{b}$ is the life time of the background spectrum collection in seconds. The percentage of the injected dose per gram (%ID/g) for different organs was calculated by dividing the activity amount of each tissue (A) to the non-decay corrected injected activity and the mass of each organ. All values were expressed as mean standard deviation, and the data were compared using Student's T-test.

***Imaging studies***

 1.85MBq of the final radioimmunoconjugate (about 100 µCi) was injected into the tumor-bearing nude mice. The mice were anesthetized, and the images were obtained.

 The patient received 1.5 mCi [^89^Zr]Zr-Trastuzumab intravenously followed by a slow intravenous infusion of unlabeled antibody to a total amount of 10 mg trastuzumab. Four days post-injection, head-to-upper thigh imaging was performed in nine-bed positions with 5 min/bed position in combination with a low dose unenhanced CT scan for attenuation correction and anatomical localization with a Biograph mCT 128-slice Siemens PET/CT system. Imaging results were compared with recently performed [^18^F]FDG PET/CT in the same patient.

## Results

***Production and quality control of*** ^89^ ***Zr***

^89^Zr was produced by the bombardment of ^89^Y_2_O_3_ pellet target using ^89^Y(p,n) ^89^Zr with specific activity of 344.1 MBq/µg and concentration of 1450.4 MBq/mL. The gamma spectrum of the ^89^Zr showed two major photons originating from ^89^Zr. The results of the radiochemical purity assessment demonstrated a radiochemical purity of higher than 99%. The amount of metal ions in the final solution was determined by the ICP-OES method.The total amount of the metal ions impurity in the final solution was less than 0.1 ppm for Yttrium as target and Aluminium as holder.

***Conjugation of DFO with trastuzumab***

 The standard curve of Arsenazo yttrium complex [Y (AAIII) 2] was employed to evaluate the average number of DFO conjugated on each trastuzumab. The data revealed that the average number of DFO coupled to trastuzumab was 0.28±0.04, and 0.41±0.09 in subsequently 3, and 10 molar excess of chelator added to the conjugation reaction. The results confirmed that by increasing the molar excess of added chelator to antibody during bioconjugation reaction, the average number of coupled chelators to each antibody is also increased. 

***Radiolabeling of DFO-Trastuzumab with*** ^89^ ***Zr***

 Different experiments considering the effects of DFO: Trastuzumab ratio, pH and time were performed to obtain the maximum radiolabeling efficiency. The best result was achieved for the chelator: mAb ratio of 10:1 at 1 h. However, radiochemical purity of about 97% was observed for all of the experiments after 24 hours.

**Figure 1:**
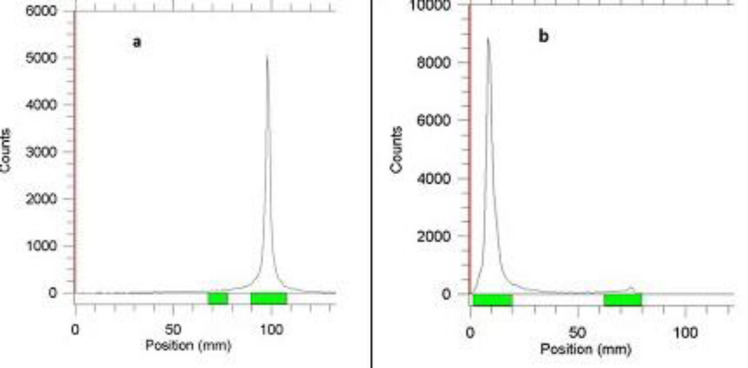
RTLC chromatogram of (a) ^89^Zr and (b) [^89^Zr]Zr-Trastuzumab using 20 mM citric acid as the mobile phase on the Whatman paper

 Also, multiple experiments indicated a radiochemical purity of higher than 98% for ^89^Zr/mAb ratio of 74 MBq: 1 mg (Figure 1). The best conditions for radiolabeling were obtained at37°C and pH=7. Finally, [^89^Zr]Zr-DFO-Trastuzumab was prepared with the labeling yield of more than 90%.

***Stability studies***

 The radiochemical purity of [^89^Zr]Zr-trastuzumab at 2, 4, 24, and 48 h after preparation in PBS buffer and human serum is indicated in Table 1. The results demonstrated that the radiolabeled complex was stable both in PBS buffer (4ºC) and freshly prepared human serum (37ºC) for at least 48 h.

**Table 1:** The radiochemical purity of [^89^Zr]Zr-Trastuzumab in PBS buffer (4°C) and human serum (37°C) at different times after preparation

**Time (h)**	**Radiochemical purity in PBS buffer (4°C)**	**Radiochemical purity in human serum (37°C)**
2	98.2%	97.9%
4	98.1%	97.9%
24	97.5%	97.7%
48	97.1%	97.1%

***Radioimmunoactivity***

 The radioimmunoactivity of the radio-immunoconjugate was studied according to the LINDMO method. The radioimmunoactivity assay of [^89^Zr]Zr-Trastuzumab demonstrated the enhancement in the binding of [^89^Zr]Zr-Trastuzumab radiopharmaceutical to HER2 receptor cells with the increment of BT474 cells, and with the number of 250×106 cells, about 70% of the [^89^Zr]Zr-Trastuzumab is bound to the HER2+ cell. Although, the binding to CHO (HER2-) cells did not differ significantly with increasing cell numbers.

***Binding Studies***

 A binding study of [^89^Zr]Zr-Trastuzumab was performed in HER2+ BT474, and HER2- CHO cells. Cell binding studies showed that about 28% of radioimmunoconjugate is attached to HER2+ BT474 cells after 90 min, which is reduced to about 22% after 72 h. The binding of ^89^Zr-DFO-Trastuzumab to HER2- CHO cells, as a control, is less than 5% both in 90 min and 72 h.

***Internalization***

 Internalization assays were carried out using BT474 and CHO cell lines to demonstrate the internalized activity as a function of time. According to the results, 50% of the radioimmunoconjugate is internalized to HER2+ BT474 cells only in 6 h post incubation with a maximum percent of the internalized activity in 8 h post incubation. Also, less than 10% of ^89^Zr-DFO-Trastuzumab is internalized in

 HER2- CHO at all intervals after incubation.

***Biodistribution studies in an animal model***

**Figure 2:**
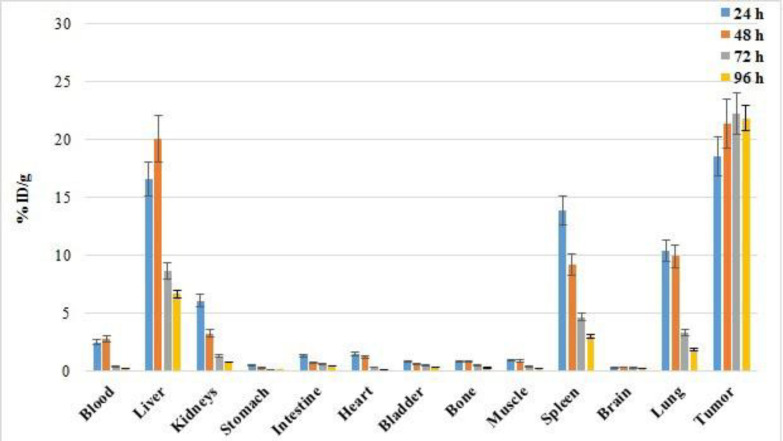
Biodistribution of [^89^Zr]Zr-Trastuzumab in tumor-bearing nude mice up to 96 h post-injection

 The biodistribution of [^89^Zr]Zr-Trastuzumab was studied in tumor-bearing nude mice. Percentages of injected activities per gram after injection of [^89^Zr]Zr-Trastuzumab is demonstrated in Figure 2.

***Imaging studies***

**Figure 3:**
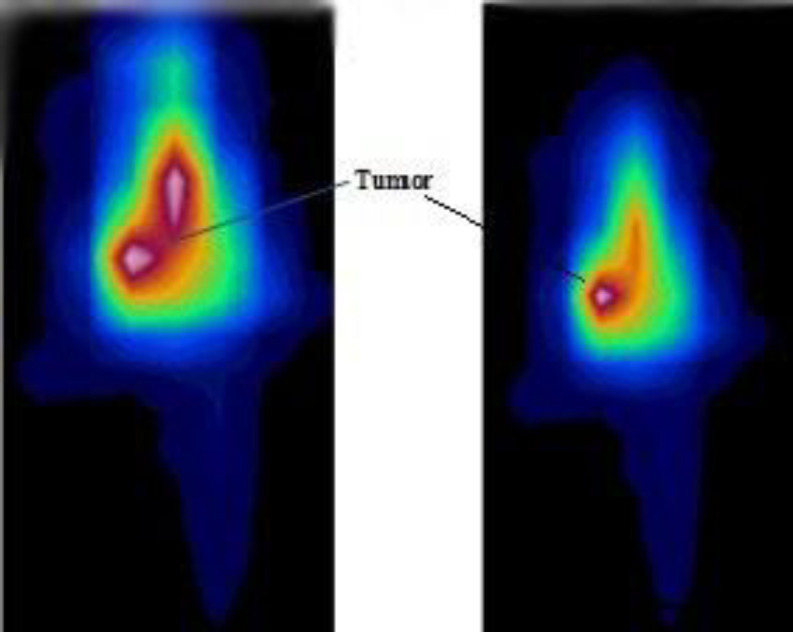
PET images of [^89^Zr]Zr-Trastuzumab in tumor-bearing mice at 24 and 72 h post-injection

 The image of tumor-bearing mice at 24 and 72 h after intravenous injection is indicated in Figure 3. Also, PET/CT images after injection of 

**Figure 4:**
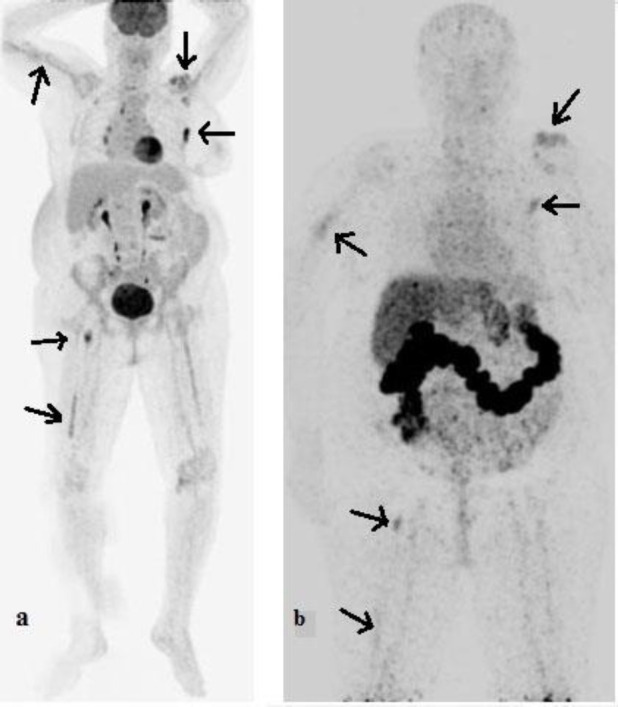
PET/CT images of a woman with HER2-positive metastatic breast cancer after injection of [^18^F]FDG (**a**) and [^89^Zr]Zr-Trastuzumab [96 h (**b**)]

[^18^F]FDG and [^89^Zr]Zr-Trastuzumab to a woman with HER2-positive metastatic breast cancer that was under treatment with Herceptin have been demonstrated in Figure 4. 

## Discussion

 Despite the promising results of [^89^Zr]Zr-trastuzumab in the diagnosis of HER2-positive tumors, according to our knowledge, no cellular studies have been reported on this radiolabeled compound towards HER2+ BT474 and CHO cell lines. In the current study, the authors tried to assess the binding, internalization, and immunoreactivity of the radioimmunoconjugate towards HER2+ BT474 and CHO cell lines.

 Some previous studies utilized a multi-step procedure with a succinylated derivative of desferrioxamine B for conjugation to trastuzumab. The optimized conditions for zirconium-trastuzumab production as well as the specific activity and radiochemical purity of the final compound presented in previously reported research are given in Table 2 for better comparison. In this study, radiolabeling of ^89^Zr with trastuzumab was performed only in two-step procedure of approximately 2 h. While several experiments were performed to achieve the best conditions for radiolabeling, the general process is similar to the chang et al. report (19). Slight modifications were made in chelator/mAb and ^89^Zr/mAb optimal ratios.

 Raava et al. study on the cell binding and internalization assay of ^89^Zr-DFO-Trastuzumab towards HER2+ SKOV-3 cells showed 2–4% binding at 1 and 4 h and approximately 4–5% at 24 h. Internalization in HER2+ SKOV-3 cells was reported at about 12–14% at 1 h and increased to 30–40% at 4 and 24 h (20). This study demonstrated the higher ability of [^89^Zr]Zr-DFO-Trastuzumab for binding to HER2+ BT474 than HER2+ SKOV-3 cells (28% at 1.5 h versus 4-5% at 24 h). A higher amount of the radiolabeled compound was also internalized to HER2+ BT474 cells (50% at 6 h versus 40% at 24 h).

**Table 2:** The optimized conditions, specific activity, and radiochemical purity of [^89^Zr]Zr-Trastuzumab

**Optimized conditions for radiolabeling**	**time**	30 min	1 h	1.5 h	1 h
	**temperature**	Room temp	37°C	37°C	37°C
	**pH**	7.4	7.0	7.1	7.0
	**Chelator/mAb molar ratio**	-	-	5:1	10:1
**Radiochemical purity investigation**	**Method/mobile phase**	ITLC/0.1 M EDTA	RTLC, 50mM DTPA	RTLC, 50mM DTPA	RTLC, 20 mM citric acid
	**percentage**	-	98.7%	≥95%	> 98%
**Specific Activity**		50 GBq/g	136.9 GBq/g	22.2-74 GBq/g	74 GBq/g
**reference**		(20)	(19)	(15)	This study

 The biodistribution study of the labeled compound in normal mice demonstrated that the liver, spleen, lungs, and kidneys had the highest uptake. The maximum uptake of the liver is observed at 4 h post-injection. 

 Biodistribution and imaging studies in tumor-bearing mice demonstrated the high uptake values of [^89^Zr]Zr-DFO-Trastuzumab in the tumor site. This biodistribution is similar to the other studies on radiolabeled antibodies (21, 22). While different studies have been performed on the biodistribution of this compound in both tumor-bearing mice and patients with breast cancer, the results of some previous studies and the current study, are presented in Table 3. Under the current research, Laforest et al. stated the liver as the dose-limiting organ (15).

**Table 3:** The results of [^89^Zr]Zr-Trastuzumab biodistribution presented in the previously reported research and the current study

**Tumor accumulation (%ID/g)**	26.2% (24 h), 93.1 % (168 h)	26.9% (24 h), 28.8% (96 h)	-	-	18.5% (24 h), 21.8% (96 h)
**Other organs with the highest accumulation (%ID/g)**	Lung, Bone marrow, Kidney	Spleen, Lung, Liver, Heart	Liver, Bone marrow, Kidney, Spleen	Liver, Kidney, Spleen	Liver, Spleen, Lung, Kidney
**Tumor/muscle ratio**	-	8.7 (24 h), 14.5 (96 h)	-	-	18.3 (24 h) 149.2 (96 h)
**Types of study**	HER2+ SKOV-3 s.c. tumor-bearing nude mice	Athymic nude mice bearing HER2+ tumors	Women with HER2-positive breast cancer	Patients with metastatic breast cancer	Nude mice bearing HER2+ tumors
**reference**	(20)	(19)	(15)	(14)	This study

Chang et al. investigation on [^89^Zr]Zr-Trastuzumab biodistribution in athymic nude mice bearing HER2+ or HER2− tumors also demonstrated the spleen, lung, liver, and heart as the organs with the most accumulation after tumor. However, the collection in the heart decreased significantly after 96 hours. 

 Dijker et al. in the first human study, observed [^89^Zr]Zr-trastuzumab biodistribution in the liver, spleen, and kidney (14). The results of the current study confirmed the previous observations; however, unlike the chang et al. study, no considerable accumulation was observed in the heart. 

 The target-to-non-target ratios at different intervals after injection of [^89^Zr]Zr-Trastuzumab is presented in Table 4. Although the radiopharmaceutical uptake into the tumor site decreases slightly after 72 h post-injection, the target-to-non-target ratios increases sharply over time. This increment is consistent with the study of chang et. al who investigated the biodistribution of [^89^Zr]Zr-trastuzumab in nude mice bearing HER2+ tumors (19). 

**Table 4:** The target to non-target ratios at different intervals after injection of [^89^Zr]Zr-Trastuzumab

**Target/non-target**	**Time**			
	**24 h**	**48 h**	**72 h**	**96 h**
Tumor/Blood	7.6	7.6	61.0	101.5
Tumor/Muscle	21.2	25.7	64.7	99.4

The tumor-to-blood ratio is increased from 7.6 (24 h) to 101.5 (96 h), and the tumor-to-muscle ratio is enhanced from 21.2 (24 h) to 99.4 (96 h). The target-to-non-target uptake ratios indicates that the PET imaging of HER2+ positive tumors can be more beneficial at higher times. Improving the imaging quality at longer times after injecting ^89^Zr-Trastuzumab is entirely under the other studies (19).

 [^89^Zr]Zr-Trastuzumab PET/CT demonstrated [^89^Zr]Zr-Trastuzumab-avid skeletal and right lung metastatic lesions, which were shown previously by [^18^F]FDG PET/CT findings. The [^18^F]FDG PET/CT scan had higher quality images. Still, the notable advantage of [^89^Zr]Zr-Trastuzumab PET/CT is delineating HER2+ metastasis, which is unique and could be significant in the diagnosis of HER2+ malignancies and HER2 based treatments.

## Conclusion

 In this study, [^89^Zr]-Trastuzumab was prepared with radiochemical purity of >98% as an immune-PET imaging agent at optimized conditions. The radioimmunoconjugate was stable both in PBS buffer and in human serum for at least 48 h. Cell studies of the radioimmunoconjugate showed a significant difference between HER2+ BT474 and HER2- CHO cell lines. The results demonstrated well radioimmunoactivity, cell binding, and internalization for [^89^Zr]Zr-Trastuzumab toward HER2+ BT474 cell lines. The biodistribution study of [^89^Zr]Zr-Trastuzumab in normal mice is entirely different from the biodistribution of free ^89^Zr. Biodistribution and imaging studies in tumor-bearing mice demonstrated the high uptake values of [^89^Zr]Zr-DFO-Trastuzumab in tumor sites. 

 The target-to-non-target uptake ratios showed that the PET imaging of HER2+ positive tumors could be more beneficial at higher times. Generally, it can be concluded that the prepared [^89^Zr]Zr-Trastuzumab has a high potential radiopharmaceutical for immune-PET imagin of patients with HER2+ tumors. [^89^Zr]Zr-Trastuzumab PET as a molecular diagnostic marker may help physicians for more accurate clinical decision-making with a non-invasive imaging technique for the assessment of HER2 status.
